# Assessing Microstructural Substrates of White Matter Abnormalities: A Comparative Study Using DTI and NODDI

**DOI:** 10.1371/journal.pone.0167884

**Published:** 2016-12-21

**Authors:** Inge Timmers, Alard Roebroeck, Matteo Bastiani, Bernadette Jansma, Estela Rubio-Gozalbo, Hui Zhang

**Affiliations:** 1 Department of Cognitive Neuroscience, Maastricht University, Maastricht, the Netherlands; 2 Department of Rehabilitation Medicine, Maastricht University, Maastricht, the Netherlands; 3 Maastricht Brain Imaging Center (M-BIC), Maastricht, the Netherlands; 4 Oxford Centre for Functional MRI of the Brain (FMRIB Centre), University of Oxford, Headington, Oxford, United Kingdom; 5 Department of Pediatrics and Laboratory Genetic Metabolic Diseases, Maastricht University Medical Center, Maastricht, the Netherlands; 6 Department of Computer Science and Centre for Medical Image Computing, University College London, London, United Kingdom; University of North Carolina at Chapel Hill, UNITED STATES

## Abstract

Neurite orientation dispersion and density imaging (NODDI) enables more specific characterization of tissue microstructure by estimating neurite density (NDI) and orientation dispersion (ODI), two key contributors to fractional anisotropy (FA). The present work compared NODDI- with diffusion tensor imaging (DTI)-derived indices for investigating white matter abnormalities in a clinical sample. We assessed the added value of NODDI parameters over FA, by contrasting group differences identified by both models. Diffusion-weighted images with multiple shells were acquired in a group of 8 healthy controls and 8 patients with an inherited metabolic disease. Both standard DTI and NODDI analyses were performed. Tract based spatial statistics (TBSS) was used for group inferences, after which overlap and unique contributions across different parameters were evaluated. Results showed that group differences in NDI and ODI were complementary, and together could explain much of the FA results. Further, compared to FA analysis, NDI and ODI gave a pattern of results that was more regionally specific and were able to capture additional discriminative voxels that FA failed to identify. Finally, ODI from single-shell NODDI analysis, but not NDI, was found to reproduce the group differences from the multi-shell analysis. To conclude, by using a clinically feasible acquisition and analysis protocol, we demonstrated that NODDI is of added value to standard DTI, by revealing specific microstructural substrates to white matter changes detected with FA. As the (simpler) DTI model was more sensitive in identifying group differences, NODDI is recommended to be used complementary to DTI, thereby adding greater specificity regarding microstructural underpinnings of the differences. The finding that ODI abnormalities can be identified reliably using single-shell data may allow the retrospective analysis of standard DTI with NODDI.

## Introduction

Diffusion-weighted imaging (DWI) can be used *in vivo* to assess properties and potential abnormalities of tissue microstructure. A variety of parameters can be estimated by measuring the diffusion of water, exploiting the fact that the diffusion is influenced by tissue microstructure. A variety of models are used to model water diffusion. Widely used–perhaps even the default model- is the single compartment diffusion tensor model [1], with fractional anisotropy (FA) as its most commonly used parameter. This straightforward marker has been studied in the context of brain development and aging [2], and has been found to be reduced in numerous neurological and neurodegenerative diseases [3,4]. Reductions in FA have been linked to axonal degeneration (e.g., in amyotrophic lateral sclerosis, ALS [5]), to myelin breakdown (e.g., in multiple sclerosis, MS [6]), or to a general state of decreased white matter integrity. Although FA is a sensitive measure, it is inherently non-specific [7]. A reduction in FA could be caused by reduced neurite density, increased dispersion of orientation, and several other factors. Related markers derived from the eigenvalues of the diffusion tensor are radial (perpendicular, d_⊥_) and axial (parallel, d_||_) diffusivity (RD and AD, respectively), and mean diffusivity (MD). It has been suggested that changes in RD reflect de/dysmyelination [8], while AD changes are more related to axonal damage [9], but the interpretation of these markers has been a topic of controversy [10].

Recently, *neurite orientation dispersion and density imaging* (NODDI) was developed to enable more specific characterisation of tissue microstructure using a clinically feasible protocol [11]. NODDI distinguishes three tissue compartments (intra-, extra-neurite, and cerebral spinal fluid—CSF) that are each modelled in a biologically informed manner, enabling several parameters to be estimated and analysed individually. Two main resulting indices are neurite density (NDI) and orientation dispersion (ODI). Measures of density and orientation dispersion in the brain have shown great correspondence to histological measures (i.e., neurite density to optical myelin staining intensity [12] and orientation dispersion to quantitative Golgi analysis [13]). Abnormalities in the morphology of neurites have been observed in diseases. For instance, axonal loss was found in MS as reflected by reductions in axonal density and area, while the WM appeared normal [14]. The correlation between FA and axonal density, however, is relatively weak. NDI, as a more specific estimate of density, might therefore be a more sensitive marker of axon pathology than FA.

*In vivo* quantification of neurite density and orientation dispersion has been shown in previous studies as well [11,15]. Recently, NODDI has been demonstrated to be useful in several applications, ranging from localisation of malformations, to characterisation of WM and GM in diseases and normal development [16–26]. Although NODDI has been thoroughly described, tested and applied, to our knowledge group inferences based on NODDI have not been explicitly compared to group inferences resulting from standard DTI. NODDI enables more specific quantification of microstructure compared to DTI, but it is very important and relevant to explicitly investigate whether this benefit manifests in a clinical study, as NODDI is potentially less sensitive due to the addition of model parameters (compared to standard DTI). Hence the value of analysing NODDI parameters has yet to be demonstrated in the context of population-based clinical studies. Therefore, the present work assesses the added value of NODDI parameters for identifying and investigating white matter abnormalities over DTI-based markers, by explicitly comparing results from NODDI and DTI analyses as applied to a clinical sample, the inherited metabolic disease classic galactosemia. In this disease, WM pathology has mainly been described in terms of diffuse signal hyperintensities on T2-weighted images [27] and has been linked at least partly to myelin abnormalities, caused by deficient galactosylation of galactocerebrosides (important building stones of myelin) [28]. The interpretation of the results in the context of the disease is published elsewhere [20]. Here, more specifically, we compared group differences using the DTI-derived (FA, RD, AD, MD) and NODDI-derived (NDI, ODI) markers and evaluated the extent to which the markers identified coinciding and unique differences in the results. By comparing DTI- and NODDI-derived group differences, this study further adds to the important practical question whether it is worthwhile to invest more imaging time to acquire multi-shell diffusion data in the context of a clinical study. In addition, we aimed to determine whether standard single-shell DTI-quality DWI data can be used for investigating white matter abnormalities based on NODDI-based tissue quantification.

## Methods

### NODDI model

NODDI allows the differentiation of three compartments in the brain–it distinguishes 1) intra-neurite space, modelled as restricted diffusion (collection of sticks forming a Watson distribution); 2) extra-neurite space, modelled as hindered, but not restricted diffusion (anisotropic Gaussian diffusion); and 3) a cerebral spinal fluid (CSF) compartment, modelled as isotropic Gaussian diffusion. The full normalised signal *A* is represented as follows: *A* = (1-*vf*_iso_) (*vf*_in_ *A*_in_ + (1-*vf*_in_) *A*_en_) + *vf*_iso_*A*_iso_, where *vf* stands for volume fraction; *in* for intra-neurite; *en* for extra-neurite; and *iso* for the isotropic CSF compartment (see [11] for a more extensive description of the model). The intra-neurite volume fraction (*vf*_in_) represents the neurite density index (NDI; typically high in WM, low in GM). The other main parameter from the NODDI estimation is the orientation dispersion index (ODI), which quantifies the angular variation of neurite orientation (ranging from 0 for perfectly coherently oriented structures to 1 for isotropic structures; typically high in GM, low in WM).

### Data acquisition

Data on eight patients with an inherited metabolic disease (classic galactosemia; see [20]) [16–21 years of age] and eight healthy controls [15–20 years of age] were acquired on a 3-T Siemens Trio whole body scanner (Siemens Medical System, Erlangen, Germany), using a 32-channel head coil. The DWI data were obtained using a double-refocused single-shot spin echo EPI sequence. 64 slices with isotropic voxels of 2.2 mm^3^ were obtained (TR = 8500 ms; TE = 97 ms) in an anterior to posterior direction. Data were acquired at two different b-values: b = 1000 s/mm^2^ with 64 diffusion-encoding gradient directions and b = 2000 s/mm^2^ with 64 diffusion directions. In addition, 5 b = 0 images were collected, two of which were acquired using a reversed phase encoding direction (posterior to anterior), to allow the estimation of susceptibility induced distortions. The diffusion encoding directions spanned the entire sphere. Total acquisition time of the DWI data was approximately 22.5 minutes. Participants were screened for MRI compatibility, and gave written informed consent (in case of minors, both parents/caregivers also gave written informed consent). The Medical Ethical Committee of the Maastricht University Hospital/Maastricht University gave ethical clearance for this study.

### Data analyses

Data pre-processing was initiated with estimation of susceptibility induced distortions. From the pairs of images acquired using reversed phase-encode directions (i.e., with distortions going in opposite directions), the susceptibility-induced off-resonance field was estimated using a method similar to the one described in Andersson et al. [29] (*topup* of FMRIB Software Library [FSL] [30]). In addition, eddy current-induced distortions and head motion were estimated, and all distortions were corrected by simultaneously modelling the effects of diffusion eddy currents (using a Gaussian process) and movements on the image (using FSL's *eddy* [31,32]). Concurrently, the b-vectors were rotated to account for the corrections (using Python; http://www.python.org).

The diffusion tensors were estimated from one shell of the corrected DWI data (b = 1000 s/mm^2^) using a linear fitting algorithm (*dtifit*, implemented in FSL). DTI-TK (publicly available; http://www.nitrc.org/projects/dtitk) was used for tensor-based spatial normalization of the volumes to an iteratively optimized population-specific template [33]. This algorithm applies a deformable registration to the tensor images, which has shown to lead to improved registration, as compared to FA-based registration algorithms [34,35]. The resulting normalized images were averaged, and high-resolution FA, RD, AD and MD maps (1 mm iso-voxel) were derived. By thinning the mean FA images, a mean FA skeleton was created that represented the centres of all tracts common to the group (tract based spatial statistics [TBSS] of FSL [36]). The aligned FA data from each subject was projected onto this skeleton using the calculated distance maps. Using the same distance maps, the RD, AD and MD maps were projected onto the FA skeleton as well. The resulting data were fed into the statistical analysis.

In parallel, *neurite orientation dispersion and density imaging* (NODDI) was applied to the pre-processed data, both on the multi-shell and on the single-shell DTI-quality (b = 1000 s/mm^2^) data (publicly available in a Matlab toolbox, http://nitrc.org/projects/noddi_toolbox). The output scalar images from NODDI (NDI, ODI, f_iso_ [CSF volume fraction], f_min_ [fitting objective function values, proportional to the fitting residuals]) were normalized to the -already defined- study-specific common group space using the transformation fields as calculated per participant during the tensor-based registration. Then, the normalized data were projected onto the -already calculated- mean FA skeleton using the original distance maps (using an adapted code from TBSS).

On the skeletonised FA, AD, RD, MD, NDI, ODI, f_iso_, f_min_ maps, permutation-based statistics were carried out (using *randomise* of FSL; 5000 permutations) using a design with group as a between-subjects factor and age as a covariate. P-values were corrected by means of the Threshold-Free Cluster Enhancement (TFCE) option [37]. A corrected alpha of 0.05 was used as the significance level.

Resulting statistical group maps were compared across measures by evaluating overlap of discriminating voxels by means of dice coefficients [2 (A ˄ B) / (A + B)] [38], and by evaluating unique contributions voxel-wise.

A pipeline of the data analysis procedure can be found in the Supporting Information (S1 File).

## Results

### NODDI revealed more specific group differences

**Fig 1:**
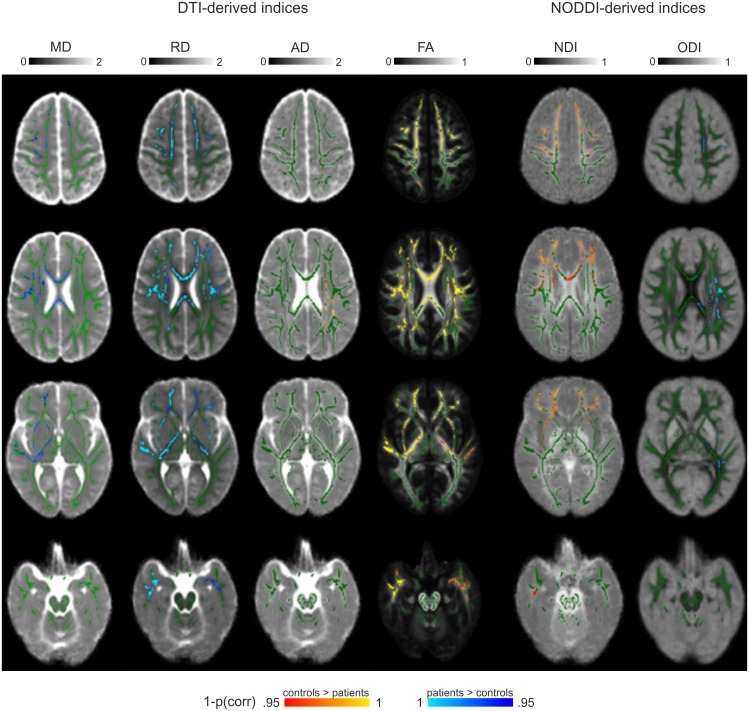
Comparison of statistical group results. Presented are the voxels discriminating across the groups by the different parameters (i.e., DTI-based: mean diffusivity [MD], axial diffusivity [AD], radial diffusivity [RD], and fractional anisotropy [FA]; NODDI-based: neurite density index [NDI], and orientation dispersion index [ODI]). A selection of slices is presented from the superior to inferior parts of the brain. In green, the mean FA skeleton is overlaid. Note that images are in radiological convention (left is right).

The FA analysis showed the most group differences, as compared to the other indices (see Fig 1 and/or Table 1). The NODDI analysis revealed several group differences in NDI and ODI that give a more specific regional pattern of white matter changes as compared to the general pattern of FA findings (Fig 1): NDI changes were found mainly in bilateral anterior regions, while ODI changes were left lateralized and more posterior (more descriptive data on the clusters can be found in [20]).

**Table 1:** Overlap in number in discriminative voxels across parameters, expressed in dice coefficients and number of voxels.

	**FA** ↓ **(23.994)** ^a^	**AD** ↓ **(1.512)**	**RD** ↑ **(19.550)**	**MD** ↑ **(6.742)**	**NDI** ↓ **(12.597)**	**ODI** ↑ **(3.283)**	**NDI+ODI (15.481)**
**FA** ↓ **(23.994)**		0.10 (1.275)	0.76 (16.578)	0.30 (4.553)	0.43 (7.770)	0.21 (2.855)	0.52 (10.236)
**AD** ↓ **(1.512)**			0.04 (425)	0 (0)	0.00 (22)	0.55 (1.319)	0.16 (1.324)
**RD** ↑ **(19.550)**				0.44 (5.720)	0.48 (7.742)	0.14 (1.641)	0.52 (9.074)
**MD** ↑ **(6.742)**					0.29 (2.768)	0.04 (175)	0.26 (2.928)
**NDI** ↓ **(12.597)**						0.05 (399)	n.a.
**ODI** ↑ **(3.283)**							n.a.
**NDI+ODI (15.481)**							

^a^ Entire FA skeleton: 82.379 voxels;

^**↑**^ = refers to increases in the patient group,

^**↓**^ = refers to decreases in the patient group;

n.a. = not applicable

### Group differences in NDI and ODI are complementary and overlap with DT indices

The group differences in NDI and ODI were complementary, supported by a minimal overlap in results (dice coefficient = 0.07). Further, the combination of NDI and ODI could explain much of the FA results, supported by a substantial overlap between the discriminative voxels identified by NODDI and FA (dice coefficient = 0.52; Table 1). Further, it can be noticed that the AD group differences overlap with ODI changes (dice coefficient = 0.55), while RD changes overlapped more with NDI changes (dice coefficient = 0.48). MD changes also overlapped more with NDI (dice coefficient = 0.29) than ODI (dice coefficient = 0.04).

### NODDI indices identify unique group differences

**Fig 2:**
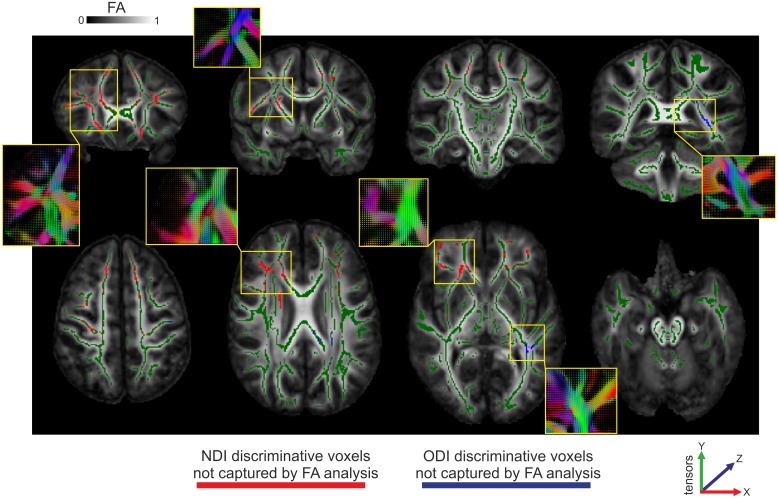
NODDI discriminative voxels not captured by FA analysis. Presented are voxels that were of discriminative value in the group NDI (red) and ODI (blue) analysis, but not in the FA analysis. Voxels are overlaid on averaged FA maps and the mean FA skeleton (green). A selection of slices is shown from anterior to posterior direction (top row), and from superior to inferior regions of the brain (bottom row). In the boxes, corresponding tensor illustrations are presented.

The NODDI parameters identified voxels discriminative across the groups that were not captured by the FA analysis: 38.3% and 13.0% of significant voxels in NDI and ODI, respectively, were not captured by the FA analysis (see Fig 2). In comparison, the AD and RD group analyses resulted in 15.7 and 15.2% not-captured-by-FA discriminative voxels, respectively.

### Checks of potential confounds

**Fig 3:**
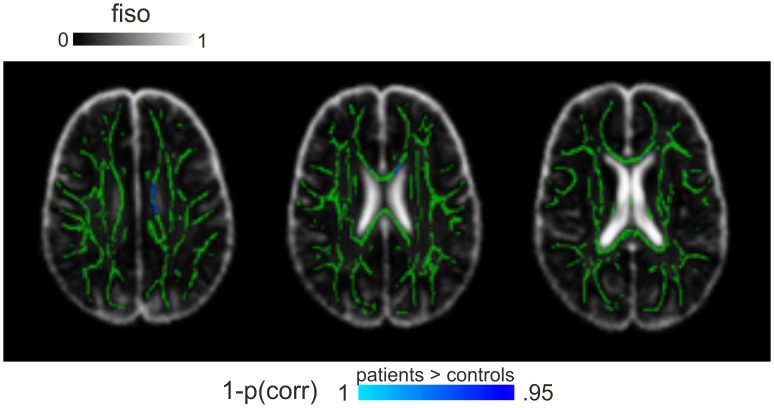
Group differences in CSF volume fraction (f_iso_). Presented are voxels that showed a significant group differences in f_iso_. Voxels are overlaid on averaged group maps. Slices are selected to optimally show the limited number of voxels showing a group difference.

Analysing the f_min_ maps, which are proportional to the fitting residuals, did not yield any significant group differences. In addition, the CSF volume fraction (f_iso_) differed only in a very small number of voxels (168 out of the 82.379 voxels of the entire WM skeleton). Here, patients showed increased f_iso_ in the body of the corpus callosum (see Fig 3).

### ODI estimations from single-shell data can be used for group inferences as well

**Fig 4:**
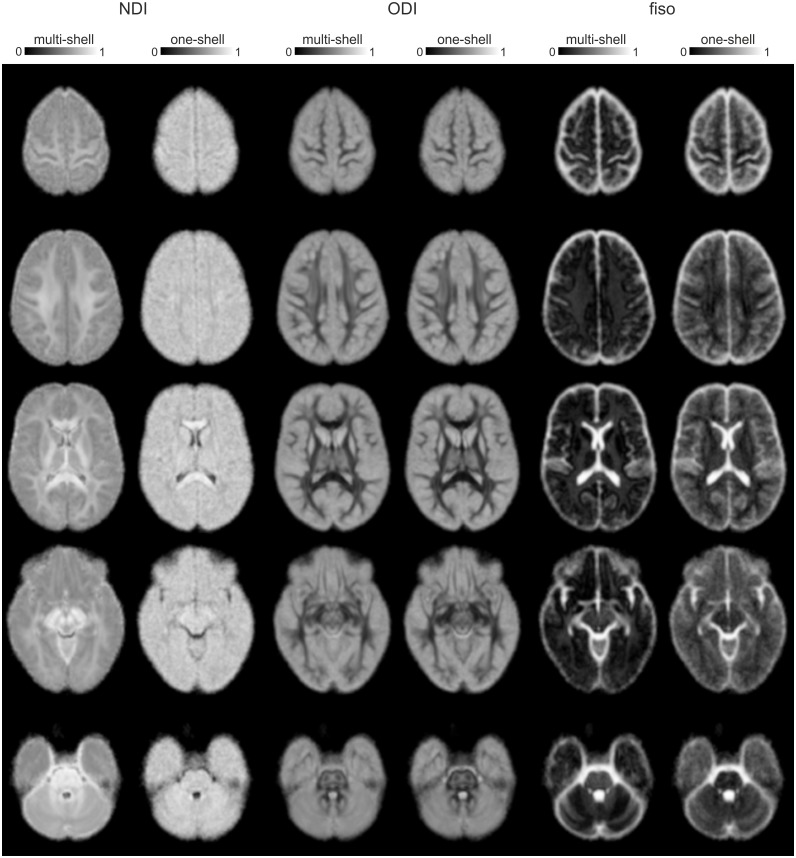
Comparison of multi- and single-shell NODDI parameter maps. Presented are the averaged NODDI parameter maps, estimated using multi-shell data, single-shell data. A selection of slices is presented from the superior to inferior parts of the brain. Visual inspection of the maps shows that ODI and f_iso_ maps are very similar across the multi- and single-shell estimations, but single-shell NDI maps are very different–more noisy–compared to multi-shell NDI maps.

**Fig 5:**
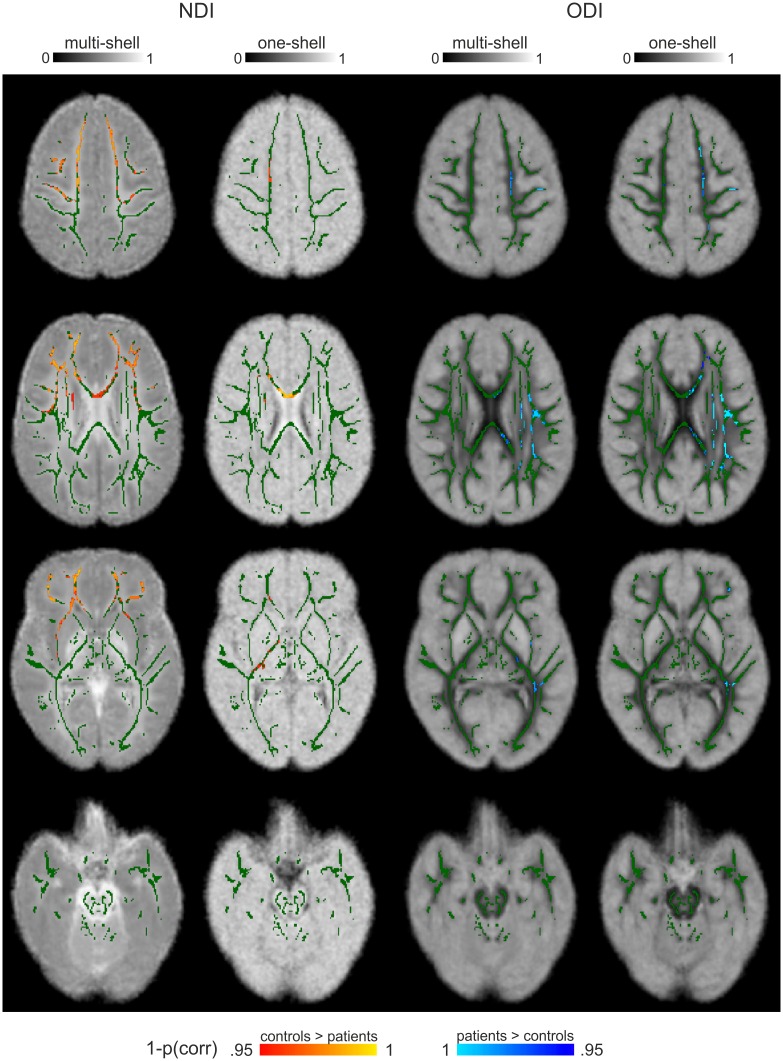
Comparison of statistical group results across multi-shell and single-shell NODDI parameter estimations. Presented are voxels that could discriminative across the groups, derived from NODDI analyses on multi- and single-shell data. A selection of slices is presented from the superior to inferior parts of the brain. In green, the mean FA skeleton is overlaid. As can be observed, there is large overlap in multi-shell and single-shell ODI discriminative voxels (dice coefficient = 0.74). Further, minimal overlap is found in NDI discriminative voxels (dice coefficient = 0.09).

A comparison between the multi-shell and single-shell fittings can be found in Fig 4, where the averaged group maps are presented. The NODDI analysis using single-shell data (b = 1000 s/mm^2^) could estimate ODI sufficiently well to be used for group inference, supported by similar ODI maps and a large overlap in the voxels discriminating across groups in single-shell and multi-shell ODI estimations (dice coefficient = 0.74) (Fig 5). NDI could not be reliably estimated using single-shell data as can be observed in the maps (e.g., no clear distinction between WM and GM), and the group results showed little overlap with the multi-shell NDI results (dice coefficient = 0.09).

## Discussion

Using a metabolic disease as an example, we demonstrated that the multi-compartment model *neurite orientation dispersion and density imaging* (NODDI) can be of added value to standard diffusion tensor imaging (DTI) for investigating WM abnormalities. NODDI reveals more specific microstructural substrates to white matter changes detected with fractional anisotropy (FA) that can be analysed independently. Also, the single-shell NODDI index of orientation dispersion (ODI) gave a very similar pattern of group differences compared to the multi-shell data.

By using a biologically informed tissue model, NODDI is capable of estimating more specific indices compared to FA: neurite density (NDI) and orientation dispersion (ODI), two key contributors to FA. In the current study, we analysed differences in the main white matter tracts across a metabolic patient group (classic galactosemia; see [20]) and a healthy control group by integrating NODDI analysis with a standard voxel-wise group inference technique TBSS. The aim was to compare overlap in group results between the standard DTI and NODDI analysis. The NODDI parameters showed little overlap in the voxels that were identified as discriminative across groups, indicating the parameters complemented each other. Taken together, however, NDI and ODI results showed a substantial overlap with FA results. In addition, we showed that these group results are not driven by NODDI model misfitting. One concern might be that there are differences in intrinsic diffusivity across groups, leading to biased estimation of the indices in one of the groups. Recently, however, it has been shown that variations in intrinsic diffusivity are reflected in the fitting residuals [39]. It is therefore important to inspect these residuals when using NODDI in group comparisons. In the current study, we did not observe any group differences in the fitting residuals, making it reasonable to conclude that intrinsic parallel diffusivity is comparable between the groups. Also, the CSF volume fraction (f_iso_) only differed minimally across groups, making it unlikely that this has biased our findings. Hence, we hereby demonstrate a conceptual disentanglement of FA into these two major contributing factors in the context of a clinical study. Further, we observed that NDI and ODI gave results that were more regionally specific compared to FA, giving more support for the idea to separately analyse these indices. Note that there is no ground truth here, but the observed regional patterns are in line with the known cognitive profile of this disease, namely higher order cognitive impairments (i.e., the anterior, bilateral profile of NDI changes), and language production and (speech) motor impairments (i.e., the predominant left-hemispheric, more posterior ODI changes; see [20] for more information on the interpretation of the results, on the clusters and correlations with behaviour) [40–43]. Furthermore, reduced NDI in this patient population is consistent with abnormal myelin associated with the disorder [27], which is linked to deficient galactosylation of galactocerebrosides (myelin building stones). From a modelling point of view, abnormal myelin increases in the extra-neurite space, which (indirectly) leads to a reduction in the (relative) volume fraction of the intra-neurite space (*vf*_in_). NDI can, however, also be affected by other processes, such as neuronal loss as this would also increase the extra-neurite space. The finding that the patients also showed increased ODI in left-lateralized regions indicates that the WM pathology is more diverse and complex than previously hypothesized. Interestingly, different brain regions reveal different WM microstructural changes, questioning which exact mechanisms underlie these findings (i.e., the left-lateralized profile of ODI fitting with motor and language problems, versus the bilateral anterior nature of NDI in line with more general higher order cognitive abnormalities). It warrants the need for further investigations to elucidate what causes these changes, and simultaneously demonstrates the added value of decomposing FA into these two separate indices to learn more about underlying pathologies.

In addition to overlap with FA findings, we observed that NDI and ODI were capable of identifying discriminative voxels that were not captured by the FA analysis. More specifically, almost 40% of the voxels that showed a significant group difference in NDI were not captured by the FA analysis, and 13% of the ODI discriminative voxels. From the location of these unique contributions (see Fig 2), it appears that this occurs at least partly in regions where there is fanning or crossing of fibres, such as in the corona radiata (but also in other regions). Previous studies have already shown that FA is weak in regions with complex fibre organisations [44]. Although NODDI does not explicitly takes crossing fibres into account, the data does suggest that NODDI analysis is of important added value in the investigation of changes in white matter microstructure in regions with more complex fibre organisations. It should be noted, however, that the FA analysis was most sensitive, or at least identified most group discriminative voxels. This could be explained by the fact that NDI and ODI each contribute to explain part of the detected FA changes, but separately they have less statistical power. It is recommended, therefore, to use both analyses in a complementary fashion.

It could be argued that radial and axial diffusivity (RD and AD, respectively) already give more specific information as compared to FA. It has been suggested that RD reflects de/dysmyelination, while AD changes reflect axonal damage [8,9]. Although the interpretation of these parameters has been discouraged in the literature [10], we made a direct comparison between these and the NODDI parameters as well. The results revealed a comparable profile with the NODDI parameters: RD and AD group results were complementary with little overlap, but together showed high overlap with FA (higher than NDI and ODI). This is not unexpected, however, as AD and RD are simply based on the eigenvalues of the diffusion tensor, and FA is computed from these same eigenvalues (and hence FA and AD/RD are not independent). In addition, NDI results showed large overlap with RD (or perpendicular diffusivity), but minimal overlap with AD (or parallel diffusivity). This was also expected, since increased neurite density would lead to decreased radial diffusivity. Further, in the NODDI tissue model, parallel diffusivity is primarily influenced by ODI [11]. Indeed, ODI results showed large overlap with parallel diffusivity (AD), but little overlap with perpendicular diffusivity (RD; for more details on the modelling aspects one can refer to [11]). It thus seems that although RD and AD give more information to complement FA, the variations can well be explained by NDI and ODI, and in a more specific and biologically informed manner. Further, RD and AD are still–like FA- based on the diffusion tensor, and thus suffer from the same weaknesses that more advanced models try to overcome using a physical model (i.e., by modelling multiple compartments, eliminating free water contamination). And, in the current study RD and AD were not as capable as NDI to capture additional discriminative voxels that FA missed. Hence, we demonstrated that NODDI parameters also have added value over the use of RD and AD.

The second main finding is that group differences in ODI could be identified reliably using standard single-shell DTI data (i.e., using one non-zero b-value in addition to the b = 0 data). The ODI maps estimated by multi-shell and single-shell (b1000) data were very comparable, as demonstrated before [11]. Further, the group results showed the same regional pattern, illustrated by a high overlap in discriminative voxels. This indicates that retrospective analysis of standard single-shell DTI data with NODDI is possible and might provide valuable additional insights on angular variation in the neurites. Note that also single shells with higher b-values can be used, as they contain higher angular resolution. Examining orientation dispersion is relevant in many respects, both in white and in grey matter. For instance, the dispersion in orientation distribution is associated with development and aging of the brain (i.e., increase and reduction, respectively), and changes in the morphology of neurites can be linked to several neurological and neurodegenerative disorders. As already demonstrated before [11], NDI could not be estimated in a reliable manner using single-shell data, as it requires both a low b-value and a high b-value shell (in addition to b = 0 images). Also in the current assessment, the maps were mainly composed of noise (i.e., no WM and GM distinction) and the group analysis did not yield any overlapping results with the multi-shell NODDI analysis.

Finally, with this study we also demonstrated the feasibility of integrating NODDI analysis with standard DWI analysis tools, such as in this case DTI-TK (for tensor-based spatial normalisation) and TBSS (for voxel-wise group inferences). The pipeline of the data analysis is available and can be found in the Supporting Information (S1 File).

To sum up, previous work has demonstrated the new insights into microstructure that NODDI can provide in a range of applications. The present study went one step further by conducting a systematic comparison between group differences determined by standard DTI and NODDI analyses. This helps clarifying the added value of NODDI analyses when making group inferences, complementary to standard DTI analysis, providing support for the adoption of a longer, multi-shell diffusion imaging protocol in clinical samples. It shows that using a clinically feasible acquisition protocol and analysis pipeline, more specific substrates of white matter (compared to DTI) can be estimated and analysed separately. The NODDI parameters complemented each other, showed little overlap, and together showed substantial overlap with FA, indicating (conceptual) disentanglement of FA into two key contributors. Results further showed that compared to FA analysis, NDI and ODI gave a pattern of results that was more regionally specific and were able to capture additional discriminative voxels that FA failed to identify. Note again that FA was still the most sensitive to group differences, as expected from the simplicity of the model, even though fitted with less data (single shell) compared to NODDI (multi-shell). NODDI therefore is recommended to be used in addition to DTI, therewith adding greater specificity. Finally, we demonstrated that retrospective analysis of the angular variation of neurites (ODI) using standard DTI-quality datasets is viable. Future analyses further need not to be limited to the WM but can extend to evaluate neurite morphology and potential changes herein in the GM as well, and could include more recent extensions of the NODDI model to include anisotropy of the orientation dispersion (Bingham-NODDI) [45].

## Supporting Information

S1 File Analysis pipeline for the TBSS analysis of DTI and NODDI data. (DOCX)
