# Supplementary material for: Assessing Microstructural Substrates of White Matter Abnormalities: A Comparative Study Using DTI and NODDI
Source: PLoS One. 2016 Dec 21;11(12):e0167884. doi: 10.1371/journal.pone.0167884 (PMC5176300; doi:10.1371/journal.pone.0167884)
Supplement: S1 File — (DOCX) [file pone.0167884.s001.docx]

**Supporting Information**

**S1 File. Analysis pipeline - TBSS analysis of DTI and NODDI data**

1. **Check data quality etc**

- Visual inspection of raw data (using *fslview* time series for instance)

1. **Pre-processing**

- Estimation of susceptibility induced distortions using pairs of b0’s acquired in opposing phase encoding directions (fsl’s *topup*). Use *fslsplit* and *fslmerge* if necessary to create a nii file with all b0’s.

*fsl5.0-topup --imain=IngTim_001_b0s --datain=acqparams.txt --config=b0.cnf --iout=IngTim_001_topup_iout --out=IngTim_001_topup_out -- fout=IngTim_001_topup_fout --logout=IngTim_001_topup_logout --verbose*

- Estimation of eddy current-induced distortions and head motion, plus correction of all distortions (fsl’s *eddy*). Combine data again and create a brain mask (fsl’s *bet* and fsl’s *fslmaths*).

*fsl5.0-eddy --imain=IngTim_001_all --mask=IngTim_001_brainmask --acqp=acqp_all_eddy.txt --index=indexfile_all.txt --bvecs=IngTim_001_all.bvec -- bvals=IngTim_001_all.bval --out=IngTim_001_all_eddy --topup=IngTim_001_topup_out –verbose*

- Rotate bvecs to account for the corrections

1. **DTI fitting using fsl dtifit**

- Fit the DTI model to the data (fsl’s *dtifit*). Note that this should be done on one shell (b1000 is more common than b2000); split the data (*fslsplit*, *fslmerge* or *fslroi*) and also adapt bvec and bval files accordingly.

*fsl5.0-dtifit --data=IngTim_001_b1k_eddy --out=IngTim_001_b1k_eddy --mask=IngTim_001_brainmask --bvecs=IngTim_001_b1k_rotated.bvec --bvals=IngTim_001_b1k.bval –verbose*

1. **Registration using DTI-TK (tensor-based registriation)**

- Download and install DTI-TK - http://dti-tk.sourceforge.net/pmwiki/pmwiki.php
- Transform data to DTI-TK format (DTI-TK’s *fsl_to_dtitk*).

*fsl_to_dtitk IngTim_001_b1k_eddy*

- Check the data using *TVtool* and *TVglyphs* (check orientation of glyphs)
- Spatial normalization in 3 steps (1. bootstrapping, 2. affine alignment, 3. diffeo alignment)

*1. Create txt file with all subject names (IngTim_001_b1k_eddy_dtitk.nii.gz etc)*

*dti_template_bootstrap /home/brain/Desktop/Tools/dtitk/ixi_aging_template_v3.0/template/ixi_aging_template.nii.gz subjs.txt*

*2. dti_affine_population mean_initial.nii.gz subjs.txt EDS 3*

*3. Create a mask - TVtool -in mean_affine3.nii.gz –tr; BinaryThresholdImageFilter mean_affine3_tr.nii.gz mask.nii.gz 0.01 100 1 0*

*dti_diffeomorphic_population mean_affine3.nii.gz subjs_aff.txt mask.nii.gz 0.002 (this last one takes a lot of time, try using an advanced computer or server)*

- Check registration by a) creating FA maps (DTI-TK’s *TVtool*) and inspecting these; b) create grids, apply transformations to the grids and inspect them using *MRIcron* for instance

1. **Post registration steps – preparation for TBSS**

- Compute the combined transformation field, and warp the data using this single transformation (one transformation is always better than multiple).

*dti_warp_to_template_group subjs.txt mean_diffeomorphic_initial6.nii 1 1 1*

- Create high resolution template from the individually warped data (*TVMean*) and generate a mean FA map from this (*TVTool*)

*TVMean -in subjs_normalized.txt -out mean_final_high_res.nii.gz (requires txt file with all normalized file names)*

*TVtool -in mean_final_high_res_masked.nii.gz –fa (rename to be consistent with TBSS: mv mean_final_high_res_masked_fa.nii.gz mean_FA.nii.gz)*

- Create FA maps for each individual and merge them into one file (*fslmerge*)

*TVtool -in IngTim_001_b1k_dtitk_diffeo.nii -fa, etc*

*fsl5.0-fslmerge -t all_FA IngTim_001_b1k_dtitk_diffeo_fa.nii ……*

- Generate the mean FA skeleton

*fsl5.0-tbss_skeleton -i mean_FA -o mean_FA_skeleton*

- Put everything in folder named in agreement with TBSS (move all_FA, mean_FA, mean_FA_mask and mean_FA_skeleton to a folder called ‘stats’)

1. **TBSS - Pre-statistics**

- Check the skeleton and see whether FA of .2 works as threshold

*fslview all_FA -b 0,0.8 mean_FA_skeleton -b 0.2,0.8 -l Green*

- Create binary skeleton mask, create distance map for projection of FA to skeleton, and create the 4D file with all skeletonized FA data – TBSS step 4

*fsl tbss_4_prestats 0.2*

1. **(For AD, RD and TR)**

- Extract the data from the normalized tensor data using TVtool

*TVtool -in IngTim_001_b1kl_dtitk_diffeo.nii.gz -ad, etc*

- Merge all data into one 4D file (*fslmerge*)
- Create mean output maps (requires txt file with file names)

*SVMean -in subjs_AD.txt -outMean mean_AD.nii.gz -outStd meanstd_AD.nii.gz*

- Copy following files to ‘stats’ folder: all_AD, mean_AD, all_FA, mean_FA, mean_FA_mask, mean_FA_skeleton, mean_FA_skeleton_mask_dst
- Project the data onto the FA skeleton using the distance map created using the FA data

*fsl5.0-tbss_skeleton -i mean_FA -p 0.2 mean_FA_skeleton_mask_dst /usr/share/fsl/5.0/data/standard/LowerCingulum_1mm all_FA all_AD_skeletonised -a all_AD -s mean_FA_skeleton*

1. **NODDI fitting (in parallel, after step 1)**

- Matlab toolbox - http://mig.cs.ucl.ac.uk/mig/mig/index.php/?n=Tutorial.NODDImatlab/
- Create a region of interest (ROI) for the NODDI fitting (usually whole brain)

*CreateROI('IngTim_001_all_eddy.nii', 'IngTim_001_brainmask.nii', 'IngTim_001_NODDI_roi.mat');*

- Convert the bval/bvec files into the required format

*protocol = FSL2Protocol('IngTim_001_all.bval', 'IngTim_001_all_rotated.bvec');*

- Create the NODDI model structure

*noddi = MakeModel('WatsonSHStickTortIsoV_B0');*

- Run the NODDI fitting (using either parallel computing toolbox or without)

*batch_fitting('NODDI_roi.mat', protocol, noddi, 'FittedParams.mat', 8); or:*

*batch_fitting_single('NODDI_roi.mat', protocol, noddi, 'FittedParams.mat');*

- Convert the estimated NODDI parameters into scalar output maps. This steps give you the following files: _ficvf.nii (NDI), _odi.nii (ODI), _fiso.nii (CSF volume fraction), _fibredirs_{x,y,z}vec.nii (fiber orientation), _fmin.nii (fitting objective function values), _kappa.nii (concentration parameter of Watson distribution used to compute ODI), and _error_code.nii (non-zero values indicate fitting errors)

*SaveParamsAsNIfTI(FittedParams, ROIFile, MaskFile, 'IngTim_001_NODDI')*

1. **Registration of the NODDI data (ficvf/NDI, ODI, fiso) to the template (after step 4)**

- Prepare NODDI output maps for DTI-TK: set origin of the scalar maps to 0 0 0

*SVAdjustVoxelspace -in IngTim_001_NODDI_ficvf.nii -origin 0 0 0 -out IngTim_001_NODDI_ficvf_origin.nii*

- Warp the data using combined transformation file (from DTI-TK) to the high resolution template

*deformationScalarVolume -in IngTim_001_NODDI_ficvf_origin.nii -trans IngTim_001_b1k_dtitk_combined.df.nii.gz -target mean_final_high_res.nii.gz -out IngTim_001_NODDI_ficvf_normalized.nii*

1. **Continue with TBSS for the NODDI data**

- Create mean output maps

*SVMean -in subjs_normalized_ficvf.txt -outMean mean_ficvf.nii.gz -outStd meanstd_ficvf.nii.gz*

- Merge the aligned data into a 4D dataset (*fslmerge*)
- Put everything in folders named in agreement with TBSS - copy files to 'stat' folder in TBSS folder, including FA files: all_NODDI_xxx, mean_NODDI_xxx, all_FA, mean_FA, mean_FA_mask, mean_FA_skeleton, mean_FA_skeleton_mask, mean_FA_skeleton_mask_dst

1. **TBSS - Pre-statistics for NODDI data**

- Skeletonise data using existing skeleton and distance map (adapted from tbss_non_FA)

*fsl5.0-tbss_skeleton -i mean_FA -p 0.2 mean_FA_skeleton_mask_dst /usr/share/fsl/5.0/data/standard/LowerCingulum_1mm all_FA all_ficvf_skeletonised -a all_ficvf -s mean_FA_skeleton*

1. **Permutation based statistics**

- Voxel-wise statistics on entire skeleton (both DTI-derived and NODDI data)

*fsl5.0-randomise -i all_ficvf_skeletonised -m mean_FA_skeleton_mask -d design.mat -t design.con -n 5000 --T2 -o stats_ficvf_tbss*
